# Recapitulation of normal collagen architecture in embryonic wounded corneas

**DOI:** 10.1038/s41598-020-70658-y

**Published:** 2020-08-14

**Authors:** Elena Koudouna, James Spurlin, Anna Babushkina, Andrew J. Quantock, James V. Jester, Peter Lwigale

**Affiliations:** 1 Gavin Herbert Eye Institute, University of California Irvine, Irvine, CA USA; 2 Structural Biophysics Research Group, School of Optometry and Vision Sciences, Cardiff University, Cardiff, Wales, UK; 3 Department of Chemical and Biological Engineering, Princeton University, Princeton, NJ USA; 4 Department of Biosciences, Rice University, Houston, TX USA

**Keywords:** Reprogramming, Regenerative medicine

## Abstract

Wound healing is characterized by cell and extracellular matrix changes mediating cell migration, fibrosis, remodeling and regeneration. We previously demonstrated that chick fetal wound healing shows a regenerative phenotype regarding the cellular and molecular organization of the cornea. However, the chick corneal stromal structure is remarkably complex in the collagen fiber/lamellar organization, involving branching and anastomosing of collagen bundles. It is unknown whether the chick fetal wound healing is capable of recapitulating this developmentally regulated organization pattern. The purpose of this study was to examine the three-dimensional collagen architecture of wounded embryonic corneas, whilst identifying temporal and spatial changes in collagen organization during wound healing. Linear corneal wounds that traversed the epithelial layer, Bowman´s layer, and anterior stroma were generated in chick corneas on embryonic day 7. Irregular thin collagen fibers are present in the wounded cornea during the early phases of wound healing. As wound healing progresses, the collagen organization dramatically changes, acquiring an orthogonal arrangement. Fourier transform analysis affirmed this observation and revealed that adjacent collagen lamellae display an angular displacement progressing from the epithelium layer towards the endothelium. These data indicate that the collagen organization of the wounded embryonic cornea recapitulate the native macrostructure.

## Introduction

Open wounds or excessive scarring arise from genetic disorders or as a result of traumatic burns or injury. Such aberrations in tissue represent a devastating source of morbidity to patients and a tremendous financial burden to the healthcare system^1–3^. Over 10 million people worldwide suffer from corneal blindness as a result of ocular trauma and infection^4,5^, whereas the total annual cost for anti-scarring treatments is estimated at $12 billion^6^. While today’s treatments including revision surgeries, generally restore structural integrity, they still struggle to overcome the natural barriers of basic wound repair process and are unable to support scarless wound healing. The promise of regenerative medicine, founded on its potential and ability to regenerate and replace tissue defects, trauma and diseases, is limited by lack of knowledge on the mechanistic basis and control between regeneration and repair. The reasons for this knowledge gap are multi-faceted, yet originating by the lack of a reliable and reproducible regenerative model, particularly for tissues with low regenerative capacities like cornea.

Tissue regeneration is a dynamic process by which damaged tissue architecture and function is restored to its normal state via cellular proliferation, differentiation, and synthesis of extracellular matrix (ECM) that matches unaffected tissue. Repair refers to a fibroproliferative response that heals damaged tissue by excessive deposition and accumulation of interstitial matrix proteins, which leads to scar formation, pathological fibrosis and loss of tissue function^7^. Scarring is, thus, a result of a reparative wound healing mechanism, disparate from the regenerative mechanism. Besides adverse aesthetic considerations and disfigurement, extensive scarring often leads to significant loss of tissue functionality, reduced quality of life and enhanced susceptibility to infection. For example, during dysfunctional healing in the skin, uncontrolled synthesis and remodeling of ECM promotes sustained inflammation, decreases tissue elasticity and reduces the natural barrier function of skin^8^. In the liver, fibrosis results in accumulation of toxins as the organ’s ability to regenerate itself and function is impaired^9^. In the cornea, scarring and fibrosis diminishes corneal transparency and leads to vision impairment and blindness^10^.

The optimal outcome of wound healing process is a regenerative wound with new tissue that is indistinguishable from the surrounding tissue. However, it is often the delicate balance between regeneration and repair that defines the result and the functionality of the tissue. While the basic mechanisms underlying the highly orchestrated wound healing process are well understood, the factors and key regulatory steps underpinning the fine-tuning of this process towards regenerative or repair (or both) pathways remain unknown^11–13^. Our understanding of factors that direct wounds to regenerate and restore normal tissue homeostasis as opposed to progressing to a chronic inflammatory and fibrotic disorder is limited. Thus, one of the major challenges of regenerative medicine is to gain knowledge that will foster the control between regeneration and repair.

Fetal wounds have the intrinsic ability to heal with a regeneration of normal tissue structure without scarring^14,15^. This scarless wound healing phenotype occurs across species but is age dependent^16–23^. An important goal of wound healing research has been to identify the cellular and molecular mechanisms that control scarless wound healing. A number of potential factors have been attributed to the scar-free regeneration of fetal wounds, including a distinct and reduced inflammatory response^24–27^ and a coordinated synthesis and remodeling of ECM^16^. Ferguson and colleagues demonstrated that transforming growth factor beta 3 (TGFβ3) is a key regulator of the scarless phenotype^29–32^. Other studies posit that the differences between scar-free healing in fetal wounds and scar-forming healing in adult wounds is related to the ECM composition and to the cell–matrix interactions^26–28^.

Collagen is the main component of tissue ECM and it plays an important role in maintaining tissue function and structural integrity. Previous studies on wound healing have brought attention to quantitative aspects of the collagen and identified differences in the ratios of collagens and in the regulation of collagen synthesis between fetal and adult wounds^33–35^. Whilst collagen fibrillogenesis is arguably one of the most important underlying mechanisms which separate scarless fetal from fibrotic adult wound repair, the regeneration of collagen and its organization after injury may be of greater importance. The organization of collagen in scarless wounds and scars is radically different, with fibrotic connective tissues lacking an organized collagen matrix^20,36^. The strength of a scar has also been shown to correlate with the covalent cross-linking of collagen^37–39^.

In the field of tissue engineering and regenerative medicine in ophthalmology, the biggest obstacle is identifying the underlying cellular and molecular mechanisms controlling regeneration versus repair. Although there are a number of clinical models that are used to evaluate the mechanisms of adult corneal wound healing, there are no models for regeneration, or recapitulation of corneal morphogenesis^40–48^. The chick cornea is anatomically and physiologically similar to the human cornea, characterized by a highly organized collagen network^49^. Although the embryonic chick cornea has been widely used to study the basis for the development of a highly organized matrix, essential for optical transparency and tissue biomechanical properties, little attention has been paid to its usage as a model for corneal regeneration^50–52^. Our previous studies, have shown that the embryonic chick cornea possess an intrinsic potential to regulate keratocyte differentiation, to restore proper innervation and rapid ECM reconstruction leading to non-fibrous restoration of the cornea^53^. Considering collagen remodeling and organization is fundamental in scarless wound healing, we asked if collagen architecture in regenerating embryonic chick corneas exhibit the normal collagen structure and morphology found in undamaged developing corneas.

In this study, we investigate the three-dimensional (3D) collagen architecture at various time points during scar-free wound healing in the embryonic chick cornea. We found that the collagen macrostructure in regenerated embryonic wounded corneas emulates the collagen architecture of the normal adult cornea^49^. Taken together, these findings combined with previous studies^53^, show that the cellular, molecular and matrix organization is efficiently recapitulated during wound healing in the embryonic chick cornea. Given the inherent difficulties in investigating fetal scarless wound healing, the embryonic chick cornea represents an excellent model to study regenerative repair. Elucidating the events and key factors that orchestrate scar-free regeneration will undoubtedly aid the development of future therapies that facilitate a healing response closer to the scarless phenotype.

## Materials and methods

### Chick embryos

Fertilized White Leghorn chick eggs were obtained from Texas A&M Poultry Center (College Station, TX, USA) and prepared according to previous protocol^54^. All experiments were approved by the Institutional Animal Care and Use Committee (IACUC) of Rice University. All animal studies were conducted in accordance with the Association for Research in Vision and Ophthalmology statement for the use of animals for ophthalmic and vision research.

### Wounding of the embryonic corneas

**Figure 1:**
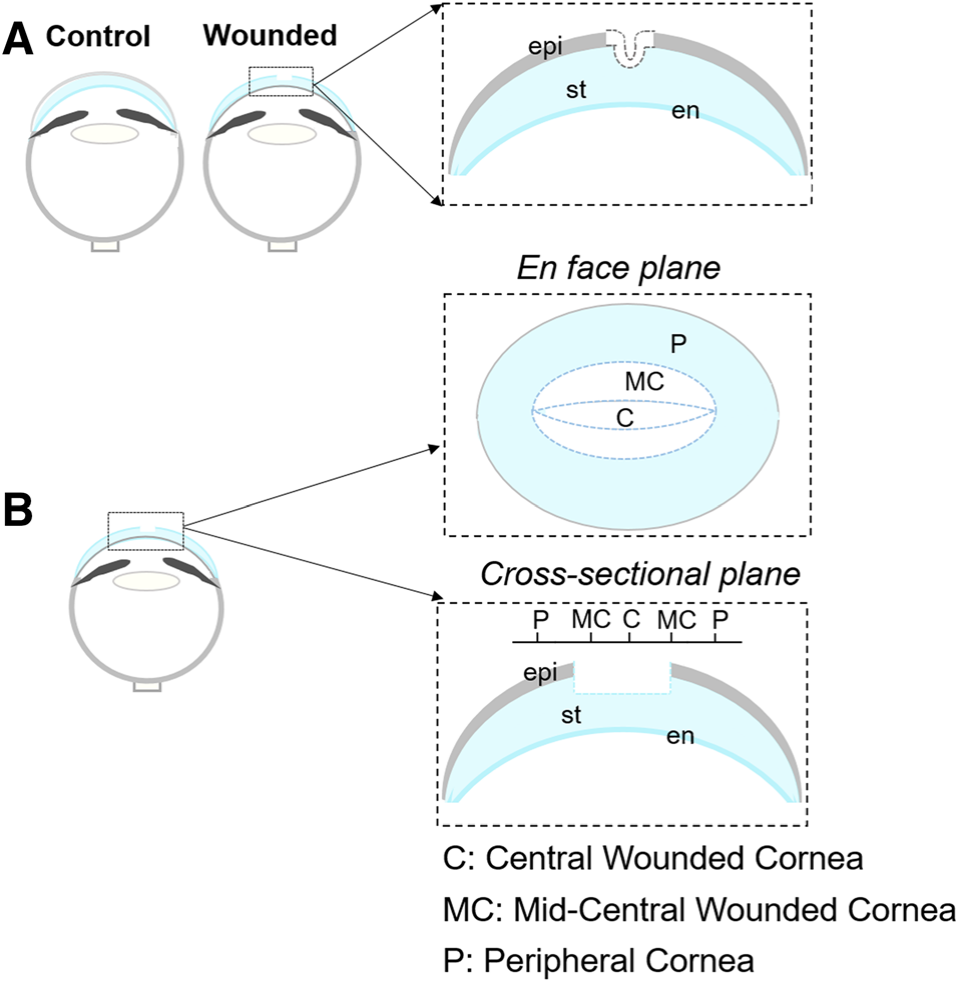
Wounding of the cornea and imaging orientation. (**A**) Schematic representation of the corneal wound. At E7, a linear incision was made traversing the corneal epithelium and anterior stroma. (**B**) The corneal wound widens as the eye grows through normal development. En face imaging of cornea was carried out from the epithelium layer towards the endothelium layer and focus datasets were obtained. Corneal cross sections were also taken and imaged through the entire tissue. The wound regions and respective nomenclature are illustrated. epi, epithelium layer; st, stroma; en, endothelium layer.

Fertilized chick eggs were incubated at 38 °C in a humidified incubator and a series of *in ovo* manipulations were carried out at embryonic day (E) 5 in order to remove the extra embryonic membranes^54^. This facilitates exposure of the embryo and access to the right eye at E7. Corneas were wounded using a micro-dissecting knife (30° Angled Micro-Dissecting Knife; Fine Science Tools, Foster City, CA) as previously described^53^. Briefly, an incision that traversed the corneal epithelium, basement membrane and anterior stroma was made across the diameter of the cornea (Fig. 1A). Such linear wounds widen to approximately one third of the corneal surface as the developing eye increases in size^53^. Three drops of Ringer’s solution containing penicillin (50 U/mL) and streptomycin (50 µg/mL) were added to embryos following wounding, and the eggs were sealed and re-incubated to obtain corneas at desired stages of wound healing. The left unwounded corneas served as control for each of the wounded corneas.

### Corneal tissue preparation

Embryos with wounded corneas were collected at 3, 5, 8, 9, 10 and 11 days post wounding (dpw). Following decapitation, eyes were collected in Ringer’s solution and fixed overnight in 4% paraformaldehyde (PFA) in phosphate-buffered saline (PBS), pH 7.2. Corneas were dissected from the surrounding scleral tissue, mounted on glass coverslips with 50% glycerol in PBS (v/v) and imaged en face for whole-mount imaging. For cross-section imaging, corneas were embedded in 10% low melting point agarose (NuSieve GTG; Lonza, Rockland, ME, USA) as previously described^52^. A schematic representation of the wounded embryonic cornea, the tissue orientation, and imaging approaches is illustrated in Fig. 1. Tissue sections of approximately 300 µm thick were cut using a vibratome (Campden Instruments Ltd.) and imaged. The wounded corneas were grouped according to the different phases of the wound healing process; early (3–5 dpw), mid (8–9 dpw) and late (10–11 dpw) healing. At least three wounded and unwounded (control) corneas were analyzed in each group.

### Second harmonic generation (SHG) microscopy

Second harmonic generation (SHG) microscopy was used to investigate the 3D organization of collagen fibrils in the wounded embryonic corneal stroma. Whole-mount and vibratome sections were imaged on a Zeiss LSM 510 (LSM 510; Carl Zeiss Inc, Thornwood, NY, USA) and a Chameleon femtosecond laser (Chameleon, Coherent Incorporated, Santa Clara, CA, USA) tuned to 820 nm. Forward and backward scattered signals were acquired using the transmitted light detector with a 430 SP filter and a band pass filter, 390/465, respectively. The samples were imaged from the epithelial surface towards the endothelial surface, using a 2 µm z-axis step size intervals to generate 3D stacks, at a resolution of 512 × 512 pixels per image and with a lateral resolution of 0.44 µm per pixel. All settings were kept constant throughout the experiments.

### Fast Fourier transform (FFT) analysis and 3D reconstruction

**Figure 2:**
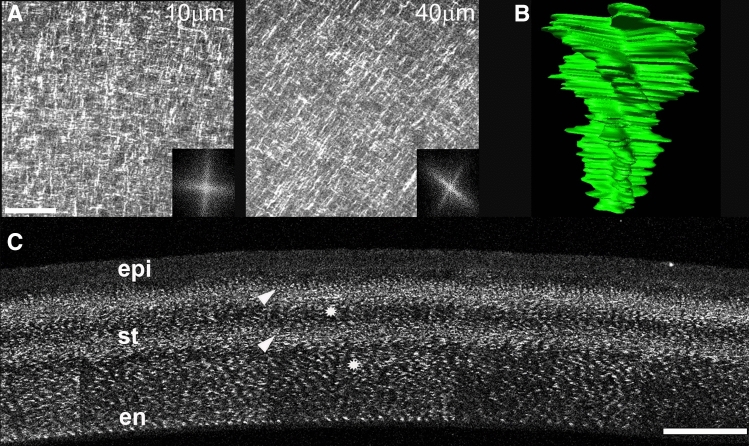
The collagen macrostructure of the chick corneal stroma is characterized by an orthogonal/rotated collagen fibril pattern. (**A**) En face SHG images taken from the epithelium layer towards the endothelium layer of E16 chick corneal stroma. The striking orthogonal/rotated collagen organization is illustrated between 10 and 40 µm depths. The respective FFT analysis of the collagen orientation are shown (insets). (**B**) Manually segmented stack of 2D FFT for the collagen throughout the corneal stroma at E16. (**C**) Cross-sectional SHG imaging of the E16 embryonic chick cornea. SHG signals are detected when collagen is aligned along the imaging plane and appear as bands (arrowheads), while collagen lamellae running “out of plane” display a weak SHG signal (asterisks). epi, Epithelium layer; st, stroma; en, Endothelium layer. Scale bars: A = 50 µm; B = 100 µm.

Collagen orientation was assessed by Fast Fourier Transform (FFT) analysis, which displays the predominant direction of collagen^55–58^. For each image plane, the FFT was generated, assembled together into a new dataset, and manually segmented. 3D reconstructed models (Figs. 2B, 5E,E′, 6B) were generated using Amira 6.4 image analysis software (Thermo Fisher Scientific, UK; https://www.thermofisher.com/co/en/home/industrial/electron-microscopy/electron-microscopy-instruments-workflow-solutions/3d-visualization-analysis-software/amira-life-sciences-biomedical.html).

## Results

### Irregular corneal stromal collagen fiber organization during the early phase of wound healing

In our previous study, we demonstrated that embryonic wounded corneas display nonfibrotic regeneration by exhibiting minimal keratocyte activation, rapid matrix regeneration and complete innervation^53^. Since collagen synthesis and subsequent organization of collagen fibers highly influence the fate of the healing wounds^58–61^, we hypothesized that the wounded embryonic corneas recapitulate the normal stromal collagen architecture.

The chick corneal stroma is built upon a remarkable collagen orthogonal/rotation paradigm^49,52^. In particular, it is composed of orthogonally aligned collagen which displays a characteristic angular displacement (rotation) through the anterior-mid stroma^49,52^. Here, we used SHG microscopy to examine the 3D collagen architecture in wounded embryonic corneas as the intrinsic nature of SHG imaging allows specific visualization of collagen and thus, precise measurement of its orientation^62,63^. This is more clearly shown in Fig. 2, which demonstrates stromal collagen organization at E16 using SHG microscopy. The orthogonal organization of collagen with angular displacement in the lamellar orientations at 10 µm and 40 µm depth is shown in en face SHG imaging (Fig. 2A). Segmentation and 3D rendering of the 2D FFT stack of collagen demonstrates the predominant collagen orientation throughout the corneal stroma (Fig. 2B). Each spoke illustrates the predominant orientation of each lamellae within that plane, while the chiral pattern highlights the angular displacement of collagen within the corneal stroma. SHG signal within cornea cross-sections is only detected when the collagen lamellae are aligned along the imaging plane (Fig. 2C, arrowheads). Therefore, regions within cornea cross-sections with low SHG signal indicate collagen fiber orientation that is rotated out of the imaging plane (Fig. 2C, asterisks). Because the collagen lamellae in the chick corneal stroma are orthogonal and display an angular displacement, longitudinally sectioned collagen fibers within corneas show a predominant banded SHG signal (Fig. 2C, arrowheads), while orthogonally arranged lamealle show a weak SHG signal (Fig. 2C, asterisks).

**Figure 3:**
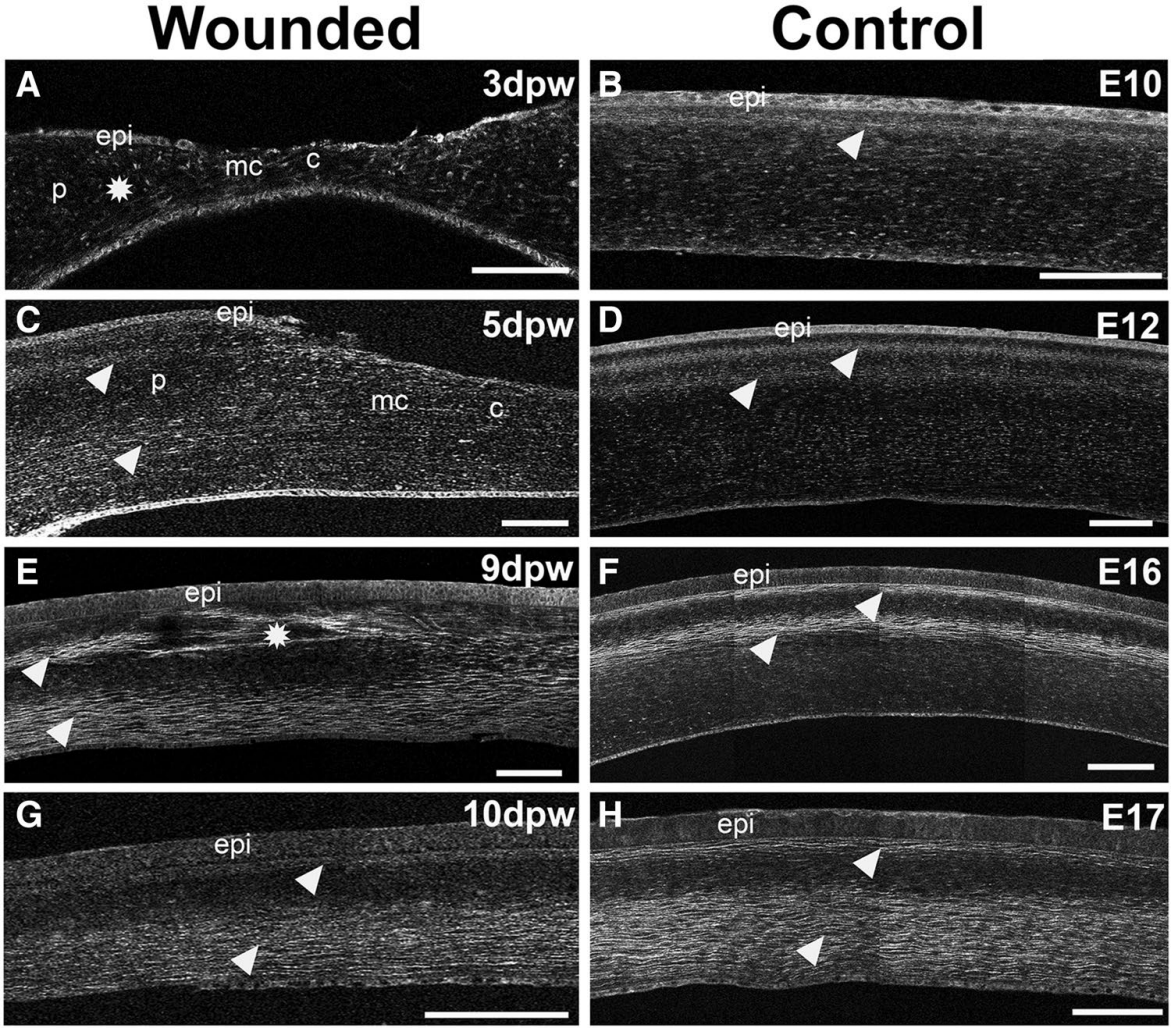
Normal collagen tissue architecture is achieved following embryonic corneal wound healing. SHG imaging of corneal cross-sections at different days post wounding (3–10 dpw) and stage-matched controls (E10–E17). (**A**) At 3 dpw, small, loosely arranged collagen fiber formation in the wounded cornea is evident during the early phase of wound healing. The irregular collagen network extended into the unwounded peripheral corneal region (**A**, asterisk). No banded SHG collagen signal is apparent indicating that the collagen layers show no angular displacement. (**B**) During normal corneal development, the angular displacement of collagen begins on E10, noted by the appearance of one band SHG signal pattern in the anterior stroma (**B**, arrowhead). (**C**, **D**) At 5 dpw, while SHG imaging did not reveal any banded SHG signal pattern at the central wounded area, the periphery of the wound displays the normal banded SHG signal pattern (**C**, arrowheads). The stage matched control showed increased angular displacement of collagen that resulted in the appearance of two banded SHG signal patterns (**D**, arrowheads). (**E**, **F**) At 9 dpw, irregular collagen network is evident in the wound region just below the corneal epithelium (**E**, asterisk). Nevertheless, two banded SHG signal patterns are evident highlighting a greater angular displacement of the collagen that are similar to stage-matched control (**E**, **F**, arrowheads). (**G**, **H**) At 10 dpw, the two banded SHG signal patterns extend throughout the corneal stroma (**G**, arrowheads), similar with the stage-matched control (**H**, arrowheads). The wound regions and corneal parts are illustrated: C, central wounded cornea; MC, mid-central wounded cornea; P, peripheral cornea; epi, epithelium layer; st, stroma; en, Endothelium layer. Scale bar: 100 µm.

Our previous investigations into the collagen fibril macrostructure during chick corneal development indicated that the complex collagen organization observed in adult corneas begins at E10, evident by the deposition of rotated collagen in the anterior stroma^52^. During development, the collagen lamellae display increased angular displacement at a greater level of stromal depth, resulting in two banded SHG signal patterns when viewed in cross-section. In our initial experiments, we wanted to determine how the corneal wounds altered the stroma by examining the 3D organization of collagen during early stages of wound healing (3–5 dpw; n = 4) by SHG microscopy. At 3 dpw, we observed a thin irregular collagen network in the central and mid-central stromal regions of the wound (Fig. 3A), which was strikingly different from the single banded SHG collagen signal of the stage-matched control (Fig. 3B, arrowhead). Surprisingly, we also observed that the irregular collagen network extended into the unwounded peripheral region of the cornea (Fig. 3A, asterisk). Cross-sectional SHG imaging did not display any banded SHG signal pattern, supporting the premise that at 3 dpw the collagen lamellae in the wound and surrounding regions show no angular displacement. Irregularly arranged collagen fibers were also observed in the stroma of the wounded cornea at 5 dpw, but they appeared bigger than those observed at 3 dpw (Fig. 3C, Supplementary Video 1). Unlike 3 dpw wounds, organization of collagen fibers in the periphery of 5 dpw wounds were similar to the two bands of SHG signal in stage-matched control, which denotes angular displacement of collagen^49,52^. In addition, contrary to control where the SHG bands appeared closer together in the anterior stroma, the second band appeared much deeper in the stroma of the wounded cornea (Fig. 3D, arrowheads). In the mid wound healing phase (8–9 dpw; n = 3), we observed disrupted collagen fiber organization in the region immediately adjacent to the corneal epithelium (Fig. 3E, asterisk). However, towards the periphery of the wound, the collagen fibers became integrated into the normal collagen bundles (Fig. 3E, arrowheads). Two SHG bands were detected in the stroma of both the wounded and stage-matched control corneas (Fig. 3E,F, arrowheads), indicating similar angular displacement of collagen. Nevertheless, the second SHG band in the wounded cornea was in the posterior stroma, opposed to the mid stroma localization in the control cornea. This finding indicates that while there are some stromal regions with disrupted collagen fibers, overall, the corneal stroma at 9 dpw is characterized by a rotational collagen lamellar organization. At the late wound healing phase (10–11 dpw; n = 3), no fragmentation or disorganization of collagen was observed. Instead, sections of 10 dpw, showed two SHG signal bands within the corneal stroma, similar to the stage-matched controls (Fig. 3G,H, arrowheads). This pattern is consistent with the complex structural organization of collagen, consisting of orthogonal lamellae with an angular displacement, in the anterior-mid stroma. Combined, our results indicate that the disrupted organization of collagen fibers in the wound re-organize during wound healing until the collagen macrostructure in the corneal stroma recapitulates the normal collagen architecture.

### Transition from random to uniaxial and biaxial collagen organization during corneal wound healing

To further examine the collagen organization during embryonic corneal wound healing, we took through-focus SHG image stacks extending from the epithelial layer towards the endothelial layer. SHG signals taken en face enable the detection of the major orientation of the collagen based on imaging of the directionality of each layer in combination with the FFT analysis^55–58^.

**Figure 4:**
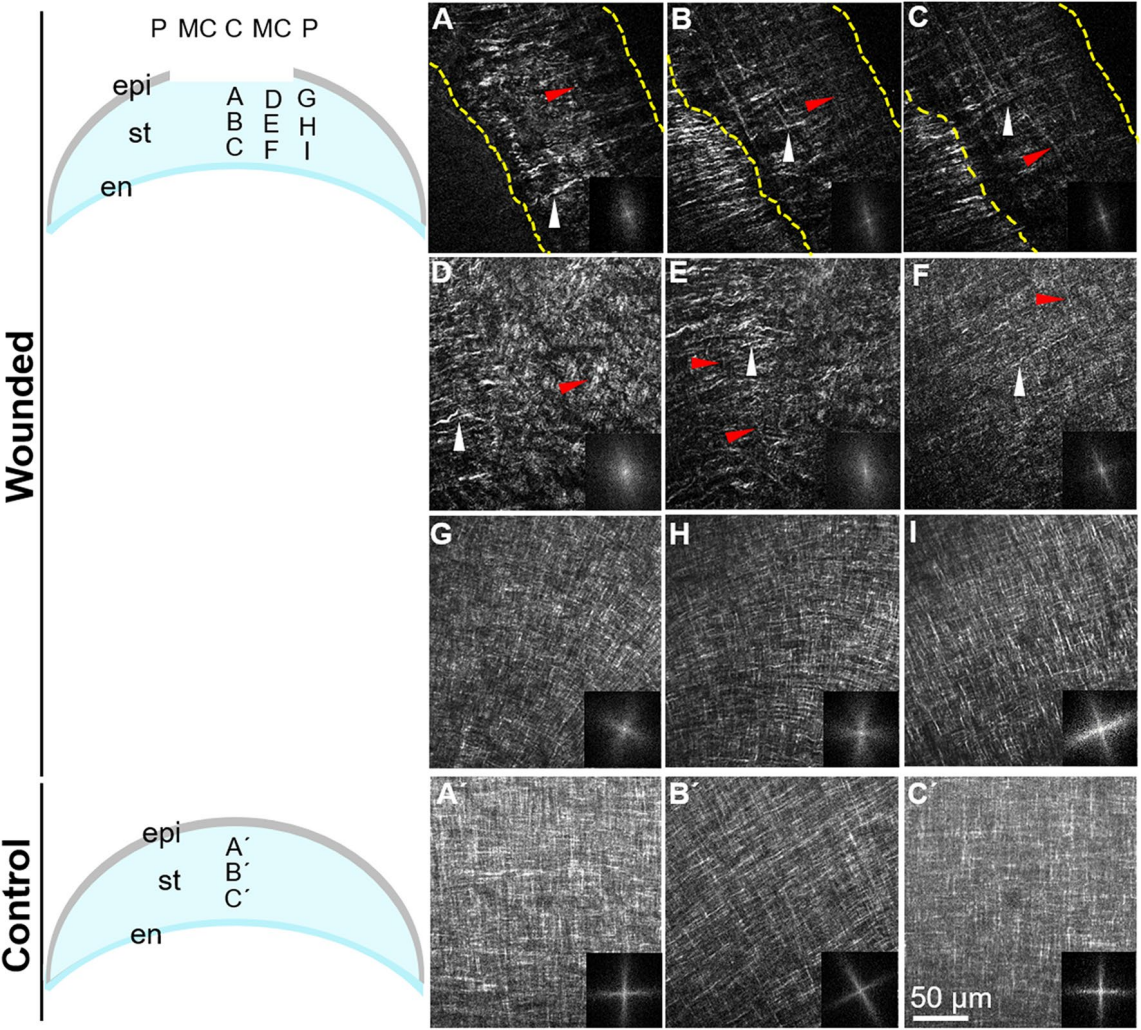
Orientation of collagen fibers bridging the corneal stroma wound cleft at 8 dpw. En face SHG images of collagen macrostructure in the central area of the wounded corneal stroma taken at successive lamellar planes, progressing from the epithelium towards the endothelium. (**A**–**C**) At the wound center, transversely (white arrowheads) and longitudinally oriented collagen fibers (red arrowheads) bridge the wound cleft, denoted by the dotted lines, at different depths of the stroma. (**D**–**F**) The mid-central wound region consists of higher density of collagen fibers compared to the central region. (**G**–**I**) The peripheral wound region is composed of normal orthogonal layers of collagen displaying a gradual angular displacement, similar to stage-matched control (**A**′–**C**′). The corresponding FFT analysis of collagen at the interface is also shown (insets). epi, epithelium layer; st, stroma; en, endothelium layer; C: central wounded cornea, MC: mid-central wounded cornea, P: peripheral cornea. Scale bar: 50 µm.

**Figure 5:**
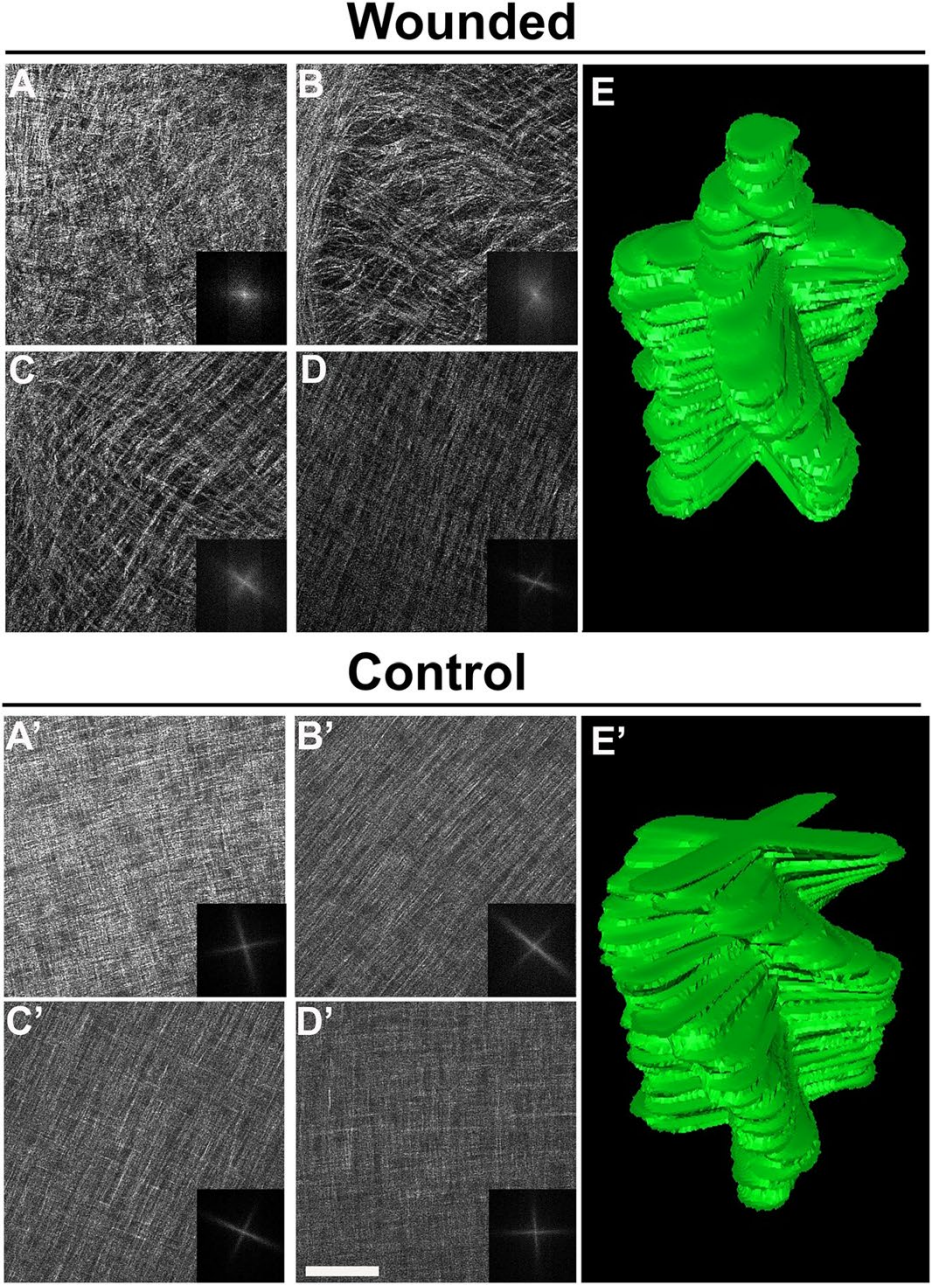
Arrangement of collagen fibers into bundles and development of angular displacement at the central wound area at 9 dpw. (**A**–**D**) En face SHG imaging microscopy and respective FFT analysis of the central wound and (**A**′–**D**′) stage-matched control at different depths of the corneal stroma, progressing from the epithelium towards the endothelium. (**E**, **E**′) 3D segmentation of 2D FFT image stacks of collagen organization throughout the corneal stroma at 9 dpw and stage-matched control. (**A**, **B**, **E**) Disorganized collagen is evident in the anterior region of the wound, below the epithelium. (**C**–**E**) In the mid-posterior wound, small bundles of collagen fibers are orthogonally arranged. The collagen lamellae at the wound region begin to develop an angular displacement. Scale bar: 50 µm.

Re-epithelialization of the embryonic corneal wounds begins after 5 dpw, and it is followed by re-establishment of normal staining patterns of stromal ECM proteins in the wound region^53^. To determine whether the organization of collagen fibers followed a similar pattern during wound healing, we analyzed their arrangement between 8 and 9 dpw (n = 3). By 8 dpw, the network of collagen fibers in the wounded area was drastically different from that observed during the early time points of wound healing. While a small degree of randomness and irregularity was observed in the central wound area, the network of collagen fibers was mostly unidirectional at different stromal depths (Fig. 4A–C, white arrowheads) (Supplementary Video 2). This pattern of organization suggests that the collagen fibers bridge the wound cleft. Occasionally, thin longitudinally oriented collagen fibers were observed in the central wound bed (Fig. 4A–C, red arrowheads). At the mid-central region of the wound, the SHG signal appeared stronger for the collagen oriented across the wound than along the wound (Fig. 4D–F, Supplementary Video 3). In the unwounded peripheral region, we observed collagen architecture with the characteristic orthogonal/rotated organization (Fig. 4G–I), reminiscent of the stage-matched control at E15 (Fig. 4A′–C′, Supplementary Video 4). At 9 dpw, the collagen fiber network in the wound changed from uniaxial into biaxial orientation. The anterior stromal region, adjacent to the overlying epithelium, at the central corneal wound, was characterized by an irregular collagen network as shown by the en face, SHG images and their corresponding FFT analysis (Fig. 5A,B,E Supplementary Video 5). Interestingly, below this region, the collagen fiber network was biaxially oriented and displayed an orthogonal lamellar orientation (Fig. 5C–E), corresponding to that of stage-matched control (Fig. 5A′–E′, Supplementary Video 6).

### Orthogonal collagen architecture with an angular displacement in re-epithelialized embryonic corneal wounds

**Figure 6:**
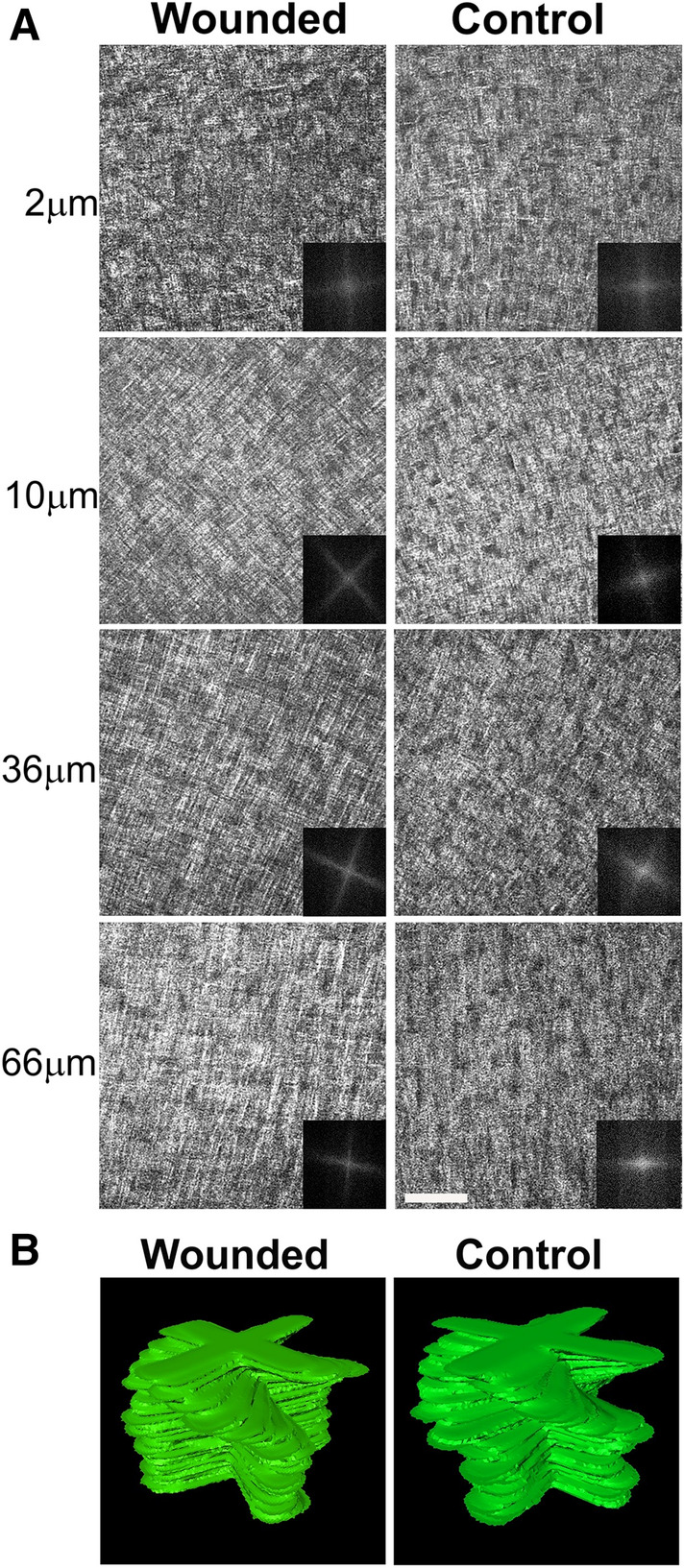
Recapitulation of the normal collagen architecture in the corneal stroma of the wound region at 10 dpw. (**A**) En face SHG images and corresponding FFT analysis (insets) of the central wound area, taken at different depths in the corneal stroma, progressing from the epithelium layer towards the endothelium layer. Bundles of collagen fibers are orthogonally organized and show an angular displacement in the anterior-mid stroma, similar with the stage-matched control. (**B**) Manually segmented stacks of 2D FFT of collagen corneal stromal organization at 10 dpw and stage-matched control. Scale bar: 50 µm.

Previously, we showed that wounded embryonic corneas are fully re-epithelialized and showed no apparent fibrosis by 11 dpw^53^. To determine whether the collagen organization returned to normal following re-epithelialization of wounded embryonic corneas, we performed SHG analysis on 10 and 11 dpw corneas (n = 3) that were fully re-epithelialized. At 10 dpw, the collagen orientation is orthogonal through the entire stroma and displays an angular displacement between adjacent lamellae, suggesting normal remodeling of collagen fiber organization associated with stromal regeneration (Fig. 6). In agreement with this finding, an orthogonal/rotated collagen fibril structural paradigm throughout the corneal stroma was also observed at 11 dpw, reminiscent of the stage-matched control at E18, indicating that during the remodeling phase of wound healing, the collagen fibers were organized in a manner to mimic normal collagen tissue architecture (Supplementary Figure 1, Supplementary Videos 7 and 8).

In our recent developmental studies of the embryonic chick cornea, a detailed analysis of collagen angular displacement from E8 to E19 showed that the angular displacement between adjacent collagen lamellae begins to first appear on E10 and gradually increases with development, showing about 193.4° by E19^52^. To better define the angular displacement of collagen and to obtain an additional quantitative measure of the level of collagen organization throughout the corneal stroma, we calculated the angular displacement in fiber directionality between adjacent lamellae at the different phase of the wound healing process. Table 1 presents the lamellar orthogonality, the degree of collagen angular displacement during the mid and late corneal wound healing phases, as well as the percentage of the rotated stroma over total stromal thickness during corneal development. No angular displacement of collagen fibers was evident at 8 dpw. The first collagen angular displacement between adjacent lamellae in the anterior stroma was observed at 9 dpw and averaged 39.41 ± 26.62°. From 9 to 11 dpw, a gradual increase in the amount of collagen angular displacement was noted; so that by 11 dpw the collagen fibers showed an angular displacement of 103.16 ± 19.21°. While the collagen angular displacement is slightly less than that measured for normal embryonic corneas in our previous studies, a similar collagen angular displacement between control and wounded eyes was observed. The reduced rotation in the current study might be due to environmental changes as a result of the embryonic manipulations required for exposing the E7 embryos in ovo. Nevertheless, the percentage of rotated stroma over the total stromal thickness increases as the cornea heals and is similar between wounded and control (71.80 ± 2.98% depth). Taken together, our results show that the organization of collagen fibers in the center of the healed wounds and throughout the corneal stroma recapitulate the normal collagen macrostructure of stage-matched unwounded corneas.

**Table 1:** Lamellar orthogonality, degree of collagen angular displacement and the percentage of rotated stroma over total stromal thickness in wounded embryonic chick corneal stroma at the mid and late wound healing phases (8–11 dpw).

	Orthogonality ± SE (°)	Angular displacement ± SE (°)	% Rotated stroma over total corneal stromal thickness ± SE
**Wounded embryonic corneas**			
Mid wound healing period (n = 3)	85.13 ± 2.53	39.41 ± 26.62	53.48 ± 4.59
Late wound healing period (n = 3)	88.13 ± 0.21	103.16 ± 19.21	71.798 ± 2.982

## Discussion

Compromised wound healing, including chronic wounds, represent a substantial burden on patients and the health system^1–3^. The balance between regeneration and tissue repair is detrimental for the restoration of tissue architecture and function after injury. Although tissue repair may restore the damaged tissue and prevent infection, it may also result in scar formation and other structural abnormalities that impair organ function. In the cornea, damage or infection often leads to fibrotic scarring, loss of transparency, and ultimately blindness^4,5^. Despite the increased knowledge of the biology of wound healing cascade in adult corneas^39–41,45,46,64–70^ and other tissues^18,24,36^, the underlying mechanisms and factors modulating the healing response in a manner favorable to regeneration have yet to be fully elucidated. The lack of sufficient regenerative models in tissues and organs with limited or no regeneration capacity, like cornea, is one of the biggest obstacles in regenerative medicine and tissue engineering.

The remarkable regenerative capacity of embryonic tissues has led to the identification of key players involved in scar-free healing^16–19,25,30–33^. The extent to which collagen synthesis, deposition, and organization takes place during wound healing highly influences the outcome and the severity of scar tissue formation^20,23,34–39^. Our previous studies showed that the embryonic chick cornea exhibits a nonfibrotic regeneration phenotype^53^. Thus, we sought to determine if the structural organization of collagen in regenerating embryonic chick corneas matches the native collagen tissue architecture. To address this, we evaluated the organization of collagen fibers in the wounded embryonic corneas at different time points following injury.

To our knowledge, we report for the first time, that the collagen macrostructure of the healed embryonic chick corneal wounds recapitulates the collagen architecture of late-stage embryonic corneas. Most importantly, these data establish the embryonic chick cornea as a valuable regeneration model and as a potential means for uncovering key factors that impact tissue regeneration over tissue repair and scar formation.

During the early phase of the wound healing response, thin collagen fibers that lack any organization were observed at the central and mid-central wound regions. This irregular network of thin collagen fibers is likely associated with the first phase of wound healing, where fibroblasts proliferate and migrate into the wound area and secrete de novo ECM, including collagen^59^. According to previous studies, the deposition of a loosely, irregular collagen network can be associated to the random organization of myofibroblasts that migrate on top of the wound bed^70^. Additionally, in evaluating the collagen content during wound healing, previous studies showed a high ratio of collagen III to total collagen in the interfibrillar space within the matrix, at the early stage of wound healing^33–35^. Based on this observation, it is possible that deposition of collagen III may lead to a loose thin disorganized collagen network similar to the arrangement noted during the early stages of wound healing in this study. Taking into account that collagen fibers are the primary load-bearing components of connective tissues, the presence of a thick irregular collagen network may also be associated with the mechanical properties of the wounded tissue, particularly, the low values of tensile strength during the early days, consistent with previous studies^58,59^.

Our results also indicate that as the synthesis and deposition of collagen increased, the orientation pattern of collagen fibers in the wounded corneal stroma drastically changed. Initially, the thin, irregular fibers were organized into a uniaxial collagen network bridging the wound cleft, likely correlating with wound contraction as measured by the reduction in wound gap at this time point during wound healing^53^. The preferential uniaxial orientation of collagen fibers across the short axis of the wound suggest that the collagen organization during healing may influence and play an important role in wound contraction. Thus, the collagen fibers may act as a bridge between the wound edges and provide resistance to tensile force. This notion is supported by previous studies which demonstrated that the arrangement of actin microfilament bundles by myofibroblasts oriented across the short axis of the wound provide the force necessary to contract the wound^69,71,72^. Taken together, these findings suggest that cell–matrix interactions, cell contractility, and possibly other internal and external forces in the wound region, may contribute to healing by inducing wound contraction.

In later stages of the cornea remodeling and regeneration, the macrostructure of collagen in the wound is characterized as a biaxial network, with collagen lamellae orthogonally oriented in the wound area. It should be noted that at this point, collagen lamellae are more compact and also begin to display angular displacement at the posterior region of the wound. This orthogonal collagen architecture is thought to guide and orient the newly deposited collagen. Since the mechanical strength of the wound develops with increasing time post wounding^59,60^, it is possible that remodeling and organization of collagen, along with levels of crosslinking and other molecules in the ECM at the wound, may additionally define the biomechanical properties of regenerating corneas. In support of this concept, the development of collagen angular displacement at the wound plays a critical role in controlling mechanical stiffness, as illustrated by our previous studies of the macrostructure of collagen in the chick corneal stroma^49,52^.

By 10 dpw, the organization of collagen lamellae detected throughout the regenerative region resembles that of normal unwounded corneal stroma. Collagen plays a fundamental role in defining the structure, function and biomechanical properties of the cornea^49^. The orthogonally organized collagen with the characteristic angular displacement of the lamellae at the anterior-mid stroma are likely involved in restoring the strength, integrity, and function of the repaired tissue^73–75^. Stromal cells have been frequently noted to influence collagen orientation^76–81^ and they are likely to also influence collagen remodeling during wound healing. It should be further noted that other ECM proteins including fibronectin, tenascin, and perlecan, are equally remodeled during the restoration of normal tissue architecture^53^. In support of this statement, Kivanany et al. recently reported the importance of cell-ECM mechanical interactions in aligned collagen deposition and organization during corneal wound healing^70^.

Despite our inability to distinguish between the native collagen from new collagen secreted post wounding, we uncovered important new insights into the 3D spatial and temporal organization of collagen during wound healing. Our results showed that the collagenous fibrillar matrix appeared to be initially organized at the mid-central wound region, then subsequently to the central wound. As the healing process takes place, collagen remodels into a normal orthogonal/rotated pattern extended from the posterior to the anterior central wound area. This further advocates that the ECM patterning and organization during the important phases of remodeling and regeneration of the healing cascade may contribute to scar-free, nonfibrotic regeneration of wounded embryonic corneas. This patterning of collagen matrix remodeling suggests an interdigitation of collagen fibers in the wound region and the surrounding tissue, which may crucially affect the wound strength and subsequent nonfibrotic wound healing. This is in agreement with previous studies which demonstrated the effects of chemoattractant gradients on fibroblasts and collagen reorientation during dermal wound healing^82–86^.

Tissue homeostasis and wound healing are intrinsically linked to the tissue’s structure and biomechanical properties^87^. In a clinical setting, the corneal biomechanical properties have been frequently noted to play a critical role in the wound healing following corneal refractive surgery and subsequently influence the predictability and visual outcomes^88,89^. Given that depth-dependent structural differences within the corneal stroma correspond to different biomechanical properties^90–93^, variations in the wound healing response among deeper wounds should also be reflected. Our data evaluating perforated corneas at 8 dpw revealed excessive accumulation of irregular collagen fibers throughout the corneal stroma, with occasional areas devoid of any collagen (Supplementary Figure 2, Supplementary Video 9), characteristic of fibrotic tissue deposition^64^. SHG imaging of fibrotic corneal wounds indicated that the disorganized fibrotic collagen fibers were interspaced with clusters of cells (Supplementary Figure 2, Supplementary Video 9), which are not observed in the sparsely populated stroma of normal corneas^49^. This advocates that under fibrotic conditions, and specifically, following corneal perforation the regenerative capacity of the embryonic cornea is lost. Hence, more comprehensive insight into the importance of wound size and depth in determining healing outcomes is needed to develop potential wound healing therapies. Furthermore, it is not yet known how the topography of the wound may impact the underlying cellular and biological activity and final healing outcome. Although biophysical and topographical cues have been increasingly recognized as key players modulating the biochemical signaling patterns associated with cell differentiation, secretion, and organization of ECM may also be involved^94–96^.

A prominent challenge in regenerative medicine is to improve wound healing repair and regeneration in adults. Research using animal model systems promises to pave the way towards more efficient and effective clinical treatments that will entice the healing and regenerative capacity of human tissues^97–99^. Embryos disclose the way to efficient repair, and the chick cornea represents an opportune model system in which the transition from fetal research to regenerative healing may be revealed^97–99^. It is important to keep in mind that despite the potential of this system to provide valuable information on the molecular and cellular mechanisms that permit scar-free wound healing, there are several differences between fetal and adult wound healing^100^ that need to be fully exploited. It is likely that tissue damage in the embryo entices repair that recapitulates morphogenesis, and the embryonic wound represents a unique milieu with distinct morphogenetic signals that are absent during adult tissue repair^100^. This concept is strengthened by recent developmental studies in zebrafish and mouse which showed that molecular cues that govern cell migration during inflammation are essential for the normal migration of germ cells^101–103^. One emphasis of future research using this model system are comparative studies between different wound healing and regeneration competence, as well as comparing healing and regeneration during embryogenesis and postnatal development. Previous studies indicated that injured adult chick corneas displayed stereotypical wound healing responses, like epithelial regeneration, neuronal re-innervation, cell proliferation and secretion of ECM molecules involved in healing^50^. In addition, the adult chick cornea was identified as an outstanding animal model to study the wound healing cascade following refractive surgery^104^. Nonetheless, there is a lack of information regarding collagen regeneration and organization following corneal wound healing in the adult chick cornea, and thus further investigations are needed to address these gaps in the field of tissue regeneration and repair. A detailed understanding of wound healing during development and maturation will provide a perceptive of how to better control the tissue’s healing and regenerative capacity.

In conclusion, the present study reports a novel finding that chick embryonic wound healing has the ability to fully recapitulate the normal 3D collagen architecture of the cornea. Given the inherent difficulties of other embryonic wound healing models, in terms of high rates of mortality and premature uterine contraction during fetal surgery, the embryonic chick cornea is an excellent and reproducible model which can serve as the primer for understanding the mechanisms controlling tissue regeneration versus repair. In addition, a detailed morphologic description of chick corneal development is already established, which allows for direct comparison with regenerating corneas. Our previous work showed that the chick corneal embryonic wounds undergo rapid regeneration leading to nonfibrotic restoration of the cornea, and therefore can serve as a model of efficient collagen synthesis and remodeling during corneal regeneration. Future investigations should be aimed at elucidating the impact of wound size, depth and location, as well as the cell–matrix interactions on the healing process. Clearly understanding the biomechanical, cellular and molecular factors that control regeneration versus repair has both clinical and scientific implications as it can lead to development of novel therapeutics, in a market of high and unmet medical need.

## Supplementary information

Supplementary Figures.

Supplementary Video 1.

Supplementary Video 2.

Supplementary Video 3.

Supplementary Video 4.

Supplementary Video 5.

Supplementary Video 6.

Supplementary Video 7.

Supplementary Video 8.

Supplementary Video 9.

## References

[1] Brown BC, McKenna SP, Siddhi K, McGrouther DA, Bayat A (2008). The hidden cost of skin scars: quality of life after skin scarring. J. Plast. Reconstr. Aesthet. Surg..

[2] Asuku ME, Ibrahim A, Ijekeye FO (2008). Post-burn axillary contractures in pediatric patients: a retrospective survey of management and outcome. Burns.

[3] Aarabi S, Longaker MT, Gurtner GC (2007). Hypertrophic scar formation following burns and trauma: new approaches to treatment. PLoS Med..

[4] Pascolini D (2004). 2002 global update of available data on visual impairment: a complication of population-based prevalence studies. Ophthalmic Epidemiol..

[5] Whitcher JP, Srinivasan M, Upadhyay MP (2001). Corneal blindness: a global perspective. Bull. World Health Organ..

[6] Sen CK (2009). Human skin wounds: a major and snowballing threat to public health and the economy. Wound Repair Regen..

[7] Atala A, Irvine DJ, Moses M, Shaunak S (2010). Wound healing versus regeneration: Role of the tissue environment in regenerative medicine. MRS Bull..

[8] Wynn TA (2008). Cellular and molecular mechanisms of fibrosis. J. Pathol..

[9] Pinzani M (2015). Pathophysiology of liver fibrosis. Dig. Dis..

[10] Cintron C, Schneider H, Kublin C (1973). Corneal scar formation. Exp. Eye Res..

[11] Tomasek JJ, Gabbiani G, Hinz B, Chaponnier C, Brown RA (2002). Myofibroblasts and mechano-regulation of connective tissue remodeling. Nat. Rev. Mol. Cell Biol..

[12] Li J, Chen J, Kirsner R (2007). Pathophysiology of acute wound healing. Clin. Dermatol..

[13] Gurtber GC, Werner S, Barrandon Y, Londaker MT (2008). Wound repair and regeneration. Nature.

[14] Burrington JD (1971). Wound healing in the fetal lamb. J. Pediatr. Surg..

[15] Rowlatt U (1979). Intrauterine wound healing in a 20 week human fetus. Virchows Arch. A Pathol. Anat. Histol..

[16] Lorenz HP (1992). Scarless wound repair: a human fetal skin model. Development.

[17] Yannas IV, Kwan MD, Longaker MT (2007). Early fetal healing as a model for adult organ regeneration. Tissue Eng..

[18] Krummel TM (1987). Fetal response to injury in the rabbit. J. Pediatr. Surg..

[19] Carter R, Sykes V, Lanning D (2009). Scarless fetal mouse wound healing may initiate apoptosis through caspase 7 and cleavage of PARP. J. Surg. Res..

[20] Beanes SR (2002). Confocal microscopic analysis of scarless repair in the fetal rat: defining the transition. Plast. Reconstr. Surg..

[21] Harsum S, Clarke JDW, Martin P (2001). A reciprocal relationship between cutaneous nerves and repairing skin wounds in the developing chick embryo. Dev. Biol..

[22] Lorenz HP, Whittby DJ, Longaker MT, Adzick NS (1993). Fetal wound healing. The ontogeny of scar formation in the non-human primate. Ann. Surg..

[23] Longaker MT (1990). Studies in fetal wound healing, VI. Second and early third trimester fetal wounds demonstrate rapid collagen deposition without scar formation. J. Pediatr. Surg..

[24] Cowin AJ, Brosnan MP, Holmes TM, Ferguson MW (1998). Endogenous inflammatory response to dermal wound healing in the fetal and adult mouse. Dev. Dyn..

[25] Hopkinson-Woolley J, Hughes D, Gordon S, Martin P (1994). Macrophage recruitment during limb development and healing in the embryonic and fetal mouse. J. Cell Sci..

[26] Larson BJ, Longaker MT, Lorenz HP (2010). Scarless fetal wound healing: a basic science review. Plast. Reconstr. Surg..

[27] Bullard KM, Longaker MT, Lorenz HP (2003). Fetal wound healing: current biology. World J. Surg..

[28] Lorenz HP, Lin RY, Longaker MT, Whitby DJ, Adzick NS (1995). The fetal fibroblast: the effector cell of scarless fetal skin repair. Plast. Reconstr. Surg..

[29] Armstrong JR, Ferguson MWJ (1995). Ontogeny of the skin and the transition from scar-free to scarring phenotype during wound healing in the pouch young of a marsupial *Monodelphis domestica*. Dev. Biol..

[30] Whitby DJ, Ferguson MWJ (1991). Immunohistochemical localization of growth factors in fetal wound healing. Dev. Biol..

[31] Occleston NL, Laverty HG, O’Kane S, Ferguson MWJ (2008). Prevention and reduction of scarring in the skin by transforming growth factor beta 3 (TGFβ3): from laboratory discovery to clinical pharmaceutical. J. Biomater. Sci. Polym. Ed..

[32] Whitby DJ, Ferguson MWJ (1991). The extracellular matrix of lip wounds in fetal, neonatal and adult mice. Development.

[33] Burd DAR, Longaker MT, Adzick NS, Harrison MR, Ehrlich HP (1990). Fetal wound healing in a large animal model: the deposition of collagen is confirmed. Brit. J. Plast. Surg..

[34] Smith LT, Holbrook KA, Madri JA (1986). Collagen types I, III, and V in human embryonic and fetal skin. Am. J. Anat..

[35] Sandberg M, Miakela JK, Multimaki P, Vuorio L, Vuorio E (1989). Construction of a human pro t I(III) collagen cDNA clone and localization of type III collagen expression in human fetal tissues. Matrix..

[36] Van Zuijlen PPM (2003). Collagen morphology in human skin and scar tissue: no adaptations in response to mechanical loading at joints. Burns.

[37] Hallok GC, Rice DC, Merkel JR, DiPaolo BR (1988). Analysis of collagen content in the fetal wound. Ann. Plast. Surg..

[38] Knight KR (1993). Collagen content of uninjured skin and scar tissue in foetal and adult sheep. Int. J. Exp. Pathol..

[39] Cintron C, Hassinger LC, Kublin CL, Cannon DJ (1978). Biochemical and ultrastructural changes in collagen during corneal wound healing. J. Ultrastruct. Res..

[40] Jester JV, Petroll WM, Barry PA, Cavanagh HD (1995). Expression of alpha-smooth muscle (alpha-SM) actin during corneal stromal wound healing. Invest. Ophthalmol. Vis. Sci..

[41] Jester JV, Barry-Lane PA, Petroll WM, Olsen DR, Cavanagh HD (1997). Inhibition of corneal fibrosis by topical application of blocking antibodies to TGF beta in the rabbit. Cornea.

[42] Saika S (2000). Role of lumican in the corneal epithelium during wound healing. J. Biol. Chem..

[43] Kamma-Lorger CS (2009). Collagen ultrastructural changes during stromal wound healing in organ cultured bovine corneas. Exp. Eye Res..

[44] Tripoli NK, Cohen KL, Proia AD (1992). Cat keratoplasty wound healing and corneal astigmatism. J. Refract. Corneal Surg..

[45] Stepp MA (2002). Defects in keratinocyte activation during wound healing in the syndecan-1-deficient mouse. J. Cell Sci..

[46] Pal-Ghosh S, Pajoohesh-Ganji A, Brown M, Stepp MA (2004). A mouse model for the study of recurrent corneal epithelial erosions: alpha9beta1 integrin implicated in progression of the disease. Invest. Ophthalmol. Vis. Sci..

[47] Aldavood SJ (2003). Effect of acetylcysteine on experimental corneal wounds in dogs. Ophthalmic Res..

[48] Gronkiewicz KM (2016). Development of a novel *in vivo* corneal fibrosis model in the dog. Exp. Eye Res..

[49] Koudouna E (2018). Evolution of the vertebrate corneal stroma. Prog. Retinal Eye Res..

[50] Ritchey ER, Code K, Zelinka CP, Scott MA, Fischer AJ (2011). The chicken cornea as a model of wound healing and neuronal re-innervation. Mol. Vis..

[51] Young RD (2014). Three-dimensional aspects of matrix assembly by cells in the developing cornea. PNAS.

[52] Koudouna E (2018). Cell regulation of collagen fibril macrostructure during corneal morphogenesis. Acta Biomater..

[53] Spurlin JW, Lwigale PY (2013). Wounded embryonic corneas exhibit nonfibrotic regeneration and complete innervation. Invest. Ophthalmol. Vis. Sci..

[54] Spurlin J, Lwigale PY (2013). A technique to increase accessibility to late-stage chick embryos for in ovo manipulations. Dev. Dyn..

[55] Russ JC, Russ JC (1995). Processing images in frequency space. The Image Processing Handbook.

[56] de Vries HJ (2000). Dermal organization in scleroderma: the fast Fourier transform and the laser scatter method objectify fibrosis in nonlesional as well as lesional skin. Lab Invest..

[57] van Zuijlen PP (2002). Morphometry of dermal collagen orientation by Fourier analysis is superior to multi-observer assessment. J. Pathol..

[58] Oxlund H, Christensen H, Seyer-Hansen M, Andreassen TT (1996). Collagen deposition and mechanical strength of colon anastomoses. J. Surg. Res..

[59] Doillon CJ, Dunn MG, Bender E, Silver FH (1985). Collagen fiber formation in repair tissue: development of strength and toughness. Collagen Relat. Res..

[60] Bailey AJ, Sims TJ, LeLouis M, Bazin A (1975). Collagen polymorphism in experimental granulation tissue. Biochem. Biophys. Res. Commun..

[61] Ehrlich HP (1994). Morphological and immunohistochemical differences between keloid and hypertrophic scar. Am. J. Pathol..

[62] Chen X, Nadiarynkh O, Plotnikov S, Campagnola PJ (2012). Second harmonic generation microscopy for quantitative analysis of collagen fibrillar structure. Nat. Protoc..

[63] Campagnola PJ (2002). Three-dimensional high-resolution second-harmonic generation imaging of endogenous structural proteins in biological tissues. Biophys. J..

[64] Farid M (2008). Detection of corneal fibrosis by imaging second harmonic-generated signals in rabbit corneas treated with mitomycin C after excimer laser surface ablation. Invest. Ophthalmol. Vis. Sci..

[65] Hassel JR, Cintron C, Kublin C, Newsome DA (1983). Proteoglycan changes during restoration of transparency in corneal scars. Arch. Biochem. Biophys..

[66] Wilson SE (2001). The corneal wound healing response: cytokine-mediated interaction of the epithelium, stroma, and inflammatory cells. Prog. Retinal Eye Res..

[67] Matsubara M, Girard MT, Kublin CL, Clinton C, Fini ME (1991). Differential roles for two gelatinolytic enzymes of the matrix metalloproteinase family in the remodeling cornea. Dev. Biol..

[68] Sundarraj N (1998). Proteoglycan distribution during healing of corneal stromal wounds in chick. Exp. Eye Res..

[69] Petroll WM, Cavanagh HD, Barry P, Andrews P, Jester JV (1993). Quantitative analysis of stress fiber orientation during corneal wound contraction. J. Cell Sci..

[70] Kivanany PB, Groser KC, Tippani M, Su S, Petroll WM (2018). Assessment of corneal stromal remodeling and regeneration after photorefractive keratectomy. Sci. Rep..

[71] Majno G, Gabbiani G, Hirschel BJ, Ryan GB, Statkov PR (1971). Contraction of granulation tissue in vitro: similarity to smooth muscle. Science.

[72] Ehrlich HP, Hunt TK (2012). Collagen organization critical role in wound contraction. Adv Wound Care (New Rochelle)...

[73] Muller L, Pels E, Vrensen G (2001). The specific architecture of the anterior stroma accounts for maintenance of corneal curvature. Br. J. Ophthalmol..

[74] Bergmanson JP, Horne J, Doughty MJ, Garcia M, Gondo M (2005). Assessment of the number of lamellae in the central region of the normal human corneal stroma at the resolution of the transmission electron microscope. Eye Contact Lens.

[75] Meek KM, Knupp C (2015). Corneal structure and transparency. Prog. Retinal Eye Res..

[76] Flynn BP, Saeidi N, Liles M, Dimarzio CA (2010). Mechanical strain stabilizes reconstituted collagen fibrils against enzymatic degradation by mammalian collagenase matrix metalloproteinase 8 (MMP-8). PLoS ONE.

[77] Jester JV (1994). Corneal keratocytes: *in situ* and *in vitro* organization of cytoskeletal contractile proteins. Invest. Ophthalmol. Vis. Sci..

[78] Eastwood M, Mudera VC, McGrouther DA, Brown RA (1998). Effect of precise mechanical loading on fibroblast populated collagen lattices: morphological changes. Cell Motil. Cytoskelet..

[79] Guido S, Tranquillo RT (1993). A methodology for the systematic and quantitative study of cell contact guidance in oriented collagen gels. Correlation of fibroblast orientation and gel birefringence. J. Cell Sci..

[80] Kim A, Lakshman N, Petroll WM (2006). Quantitative assessment of local collagen matrix remodeling in 3-D culture: the role of Rho kinase. Exp. Cell Res..

[81] Karamichos D, Lakshman N, Petroll WM (2007). Regulation of corneal fibroblast morphology and collagen reorganization by extracellular matrix mechanical property. Invest Ophthalmol. Vis. Sci..

[82] McDougall S, Dallon J, Sherratt J, Maini P (2006). Fibroblast migration and collagen deposition during dermal wound healing: mathematical modelling and clinical implications. Philos. Trans. Math. Phys. Eng. Sci..

[83] Ferguson MWJ, O’Kane S (2004). Scar-free healing: from embryonic mechanisms to adult therapeutic intervention. Philos. Trans. R. Soc. Lond. B Biol. Sci..

[84] Taya Y, O’Kane S, Ferguson MWJ (1999). Pathogenesis of cleft palate in TGF-b3 knockout mice. Development.

[85] Dallon JC, Sherratt JA, Maini PK, Ferguson MWJ (2000). Biological implications of a discrete mathematical model for collagen deposition and alignment in dermal wound repair. IMA J. Math. Appl. Med. Biol..

[86] Dallon JC, Sherratt JA, Maini PK (2001). Modeling the effects of transforming growth factor-b on extracellular matrix alignment in dermal wound repair. Wound Repair Regen..

[87] Gurtner GC (2011). Improving cutaneous scar formation by controlling the mechanical environment: large animal and phase I studies. Ann. Surg..

[88] Roberts C (2002). Biomechanics of the cornea and wavefront-guided laser refractive surgery. J. Refract. Surg..

[89] Dupps WJ, Wilson SE (2006). Biomechanics and wound healing in the cornea. Exp. Eye Res..

[90] Winkler M (2011). Nonlinear optical macroscopic assessment of 3-D corneal collagen organization and axial biomechanics. Invest. Ophthalmol. Vis. Sci..

[91] Randleman JB, Dawson DG, Grossniklaus HE, McCarey BE, Edelhauser HF (2008). Depth-dependent cohesive tensile strength in human donor corneas: implications for refractive surgery. J. Refract. Surg..

[92] Kohlhaas M (2006). Biomechanical evidence of the distribution of cross-links in corneas treated with riboflavin and ultraviolet A light. J. Cataract Refract. Surg..

[93] Scarcelli G (2013). Brillouin microscopy of collagen crosslinking: noncontact depth-dependent analysis of corneal elastic modulus. Invest. Ophthalmol. Vis. Sci..

[94] Guo X (2007). Morphological characterization of organized extracellular matrix deposition by ascorbic acid-stimulated human corneal fibroblasts. Invest. Ophthalmol. Vis. Sci..

[95] Saeidi N (2012). Disorganized collagen scaffold interferes with fibroblast mediated deposition of organized extracellular matrix in vitro. Biotechnol. Bioeng..

[96] Zareian R (2016). Human corneal fibroblast pattern evolution and matrix synthesis on mechanically biased substrates. Tissue Eng..

[97] Redd MJ, Cooper L, Wood W, Stramer B, Martin P (2004). Wound healing and inflammation: embryos reveal the way to perfect repair. Philos. Trans. R Soc. Lond..

[98] Degen KE, Gourdie RG (2012). Embryonic wound healing: a primer for engineering novel therapies for tissue repair. Birth Defects Res. C Embryo Today.

[99] Jingjing L, Siwei Z, Enrique A (2016). The cellular and molecular mechanisms of tissue repair and regeneration as revealed by studies in Xenopus. Regeneration.

[100] Martin P, Parkhurst SM (2004). Parallels between tissue repair and embryo morphogenesis. Development.

[101] Doitsidou M (2002). Guidance of primordial germ cell migration by the chemokine SDF-1. Cell.

[102] Knaut H, Werz C, Geisler R, Nusslein-Volhard C (2003). A zebrafish homologue of the chemokine receptor Cxcr4 is a germ-cell guidance receptor. Nature.

[103] Molyneaux KA (2003). The chemokine SDF1/CXCL12 and its receptor CXCR4 regulate mouse germ cell migration and survival. Development.

[104] Fowler WC, Chang DH, Roberts BC, Zarovnaya EL, Proia AD (2004). A new paradigm for corneal wound healing research: the white leghorn chicken (*Gallus gallus domesticus*. Curr. Eye Res..

