# Supplementary material for: Recapitulation of normal collagen architecture in embryonic wounded corneas
Source: Sci Rep. 2020 Aug 14;10:13815. doi: 10.1038/s41598-020-70658-y (PMC7427794; doi:10.1038/s41598-020-70658-y)
Supplement: Supplementary file 1 — Supplementary Figures. [file 41598_2020_70658_MOESM1_ESM.docx]

**Recapitulation of normal collagen architecture in embryonic wounded corneas**

Elena Koudouna, James Spurlin, Anna Babushkina, Andrew J. Quantock, James V. Jester, Peter Lwigale

**Supplementary Material**

**Supplementary Figure 1**


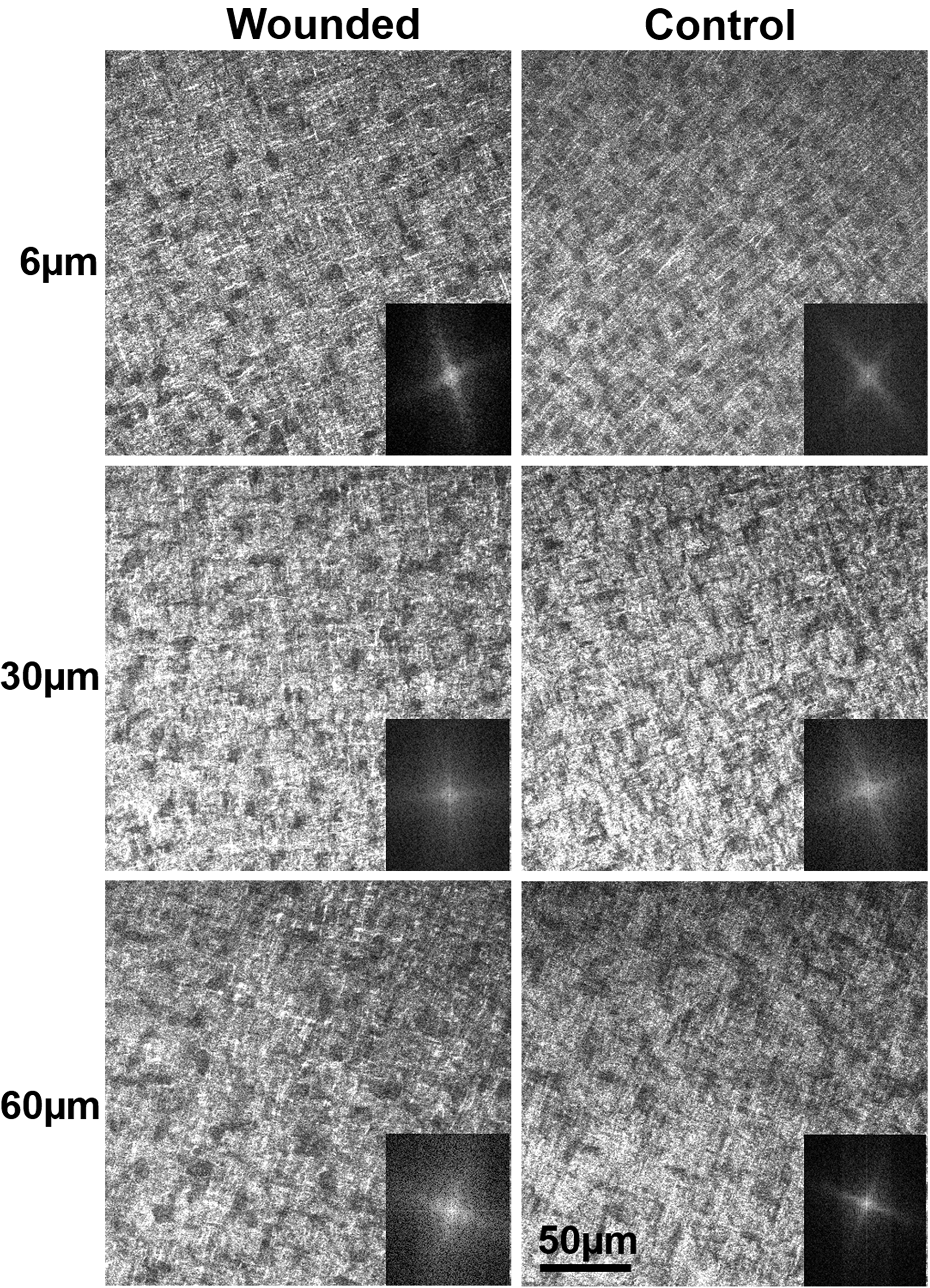


**Supplementary Figure 1:** Normal collagen architecture in the corneal stroma of the wound region by 11dpw. En face SHG images of 11dpw and stage-matched E18 control cornea taken at successive lamellar planes, progressing from the epithelium towards the endothelium layer. The corresponding FFT analysis at the interface is shown (insets). Scale bar: 50 µm

**Supplementary Figure 2**


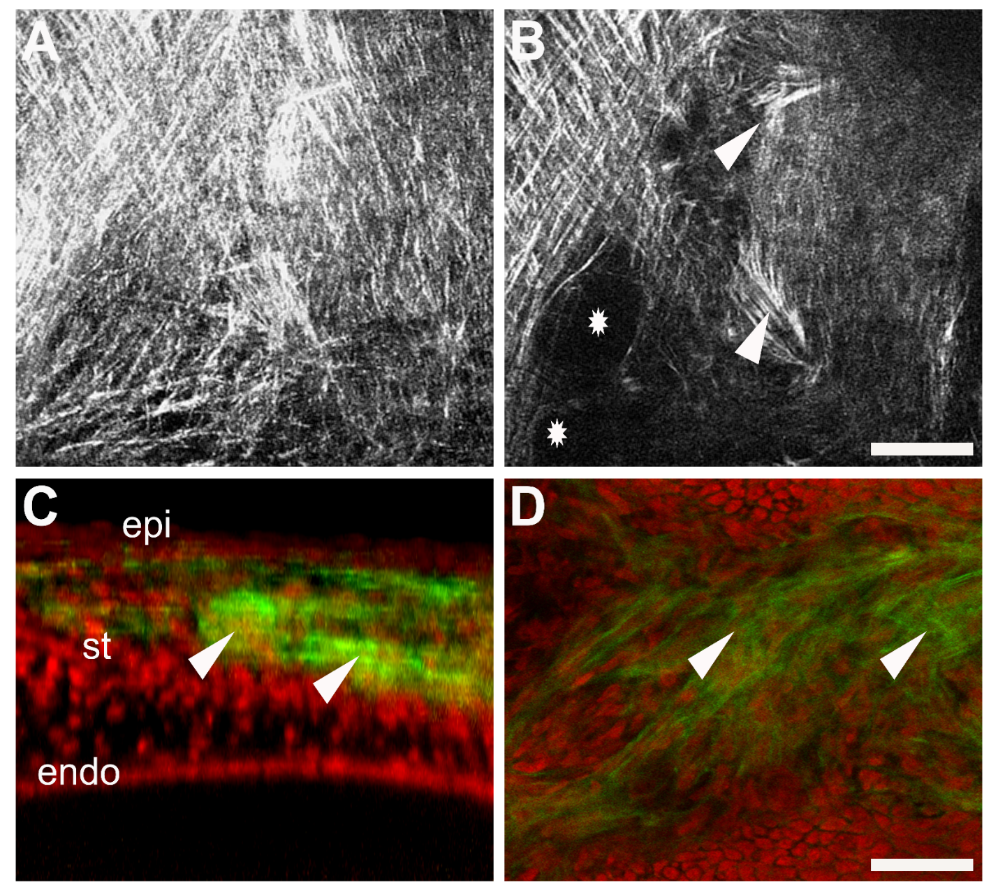


**Supplementary Figure 2:** Perforation of the embryonic chicken cornea induces a fibrotic response and a disorganized collagen network. (A) Maximum intensity projection image of a 140 µm-thick SHG dataset at 8 dpw. (B) En face SHG image of the perforated cornea going from the epithelium layer towards the endothelium layer showing irregular, disorganized collagen and stromal regions of fibrosis (arrowheads). Areas devoid of collagen are also observed (asterisks). Scale bar: 50 µm. (C) XZ maximum intensity projection image of a confocal fluorescence dataset of the perforated corneal stroma showing FITC phalloidin staining of actin filaments (green) and propidium iodide staining of cell nuclei (red). Fibrotic scar is evident in the anterior-mid stroma (arrowheads). (D) En face fluorescence image of the anterior stroma showing a region of fibrosis associated with abnormal, abundant actin organization (arrowheads). Scale bar: 50 µm. Epi; epithelium layer, St; stroma, Endo; endothelium layer.

**
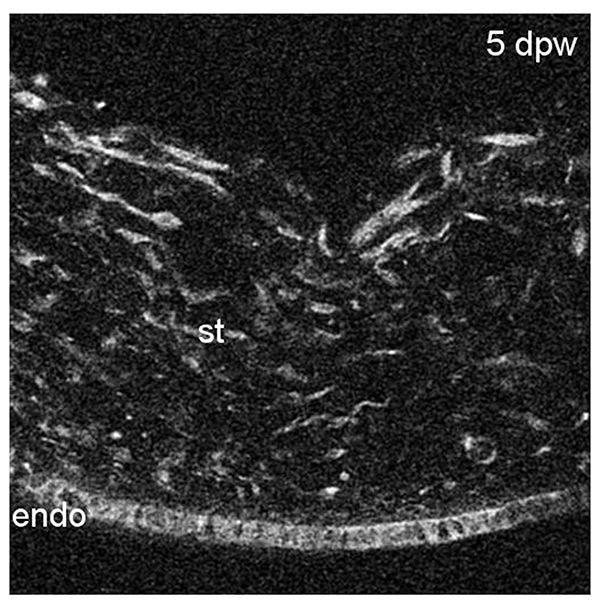
**

**Supplementary Video 1:** SHG collagen dataset of a corneal cross-section focused at the central wounded region at 5dpw. St: stroma; endo: endothelium.

**
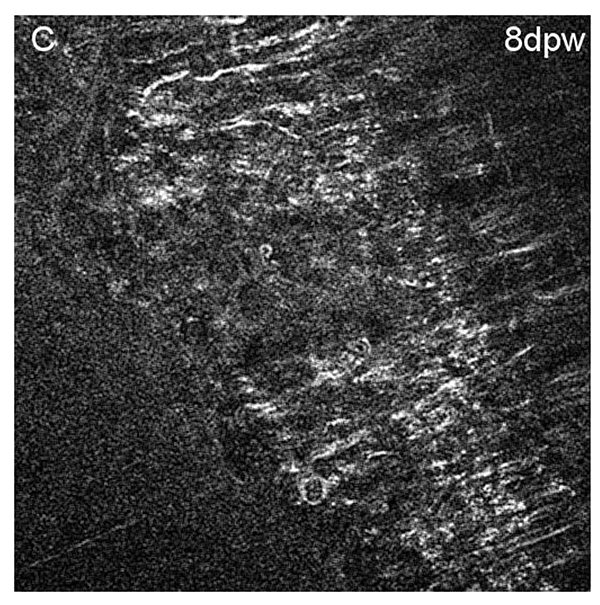
**

**Supplementary Video 2:** Through-focus SHG collagen dataset of the collagen organization at the central wound (C) at 8dpw.


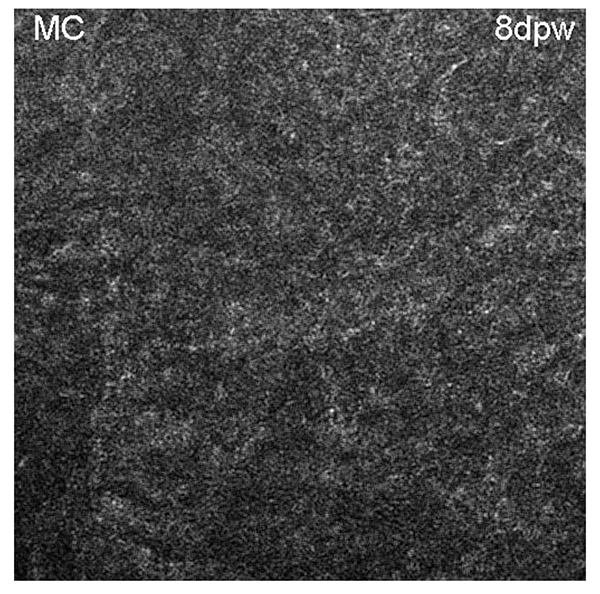


**Supplementary Video 3:** Through-focus SHG collagen dataset of the collagen organization at the mid-central wound (MC) at 8dpw.

**
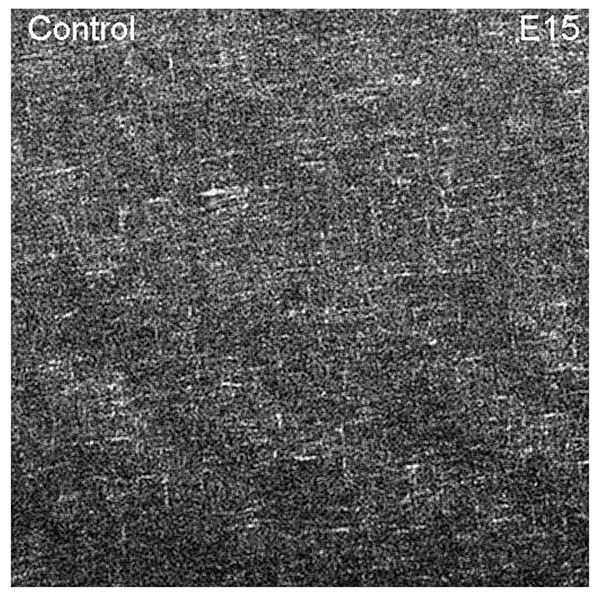
**

**Supplementary Video 4:** Through-focus SHG collagen dataset of the collagen organization, going from the epithelium layer towards the endothelium layer, in the embryonic corneal stroma at E15.


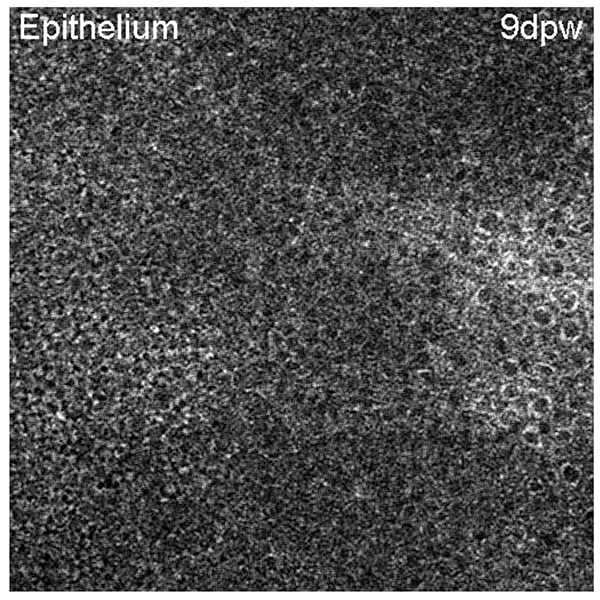


**Supplementary Video 5:** Through-focus SHG collagen dataset of the collagen organization, going from the epithelium layer towards the endothelium layer, at the central wound (C) at 9dpw. Epi: epithelium.


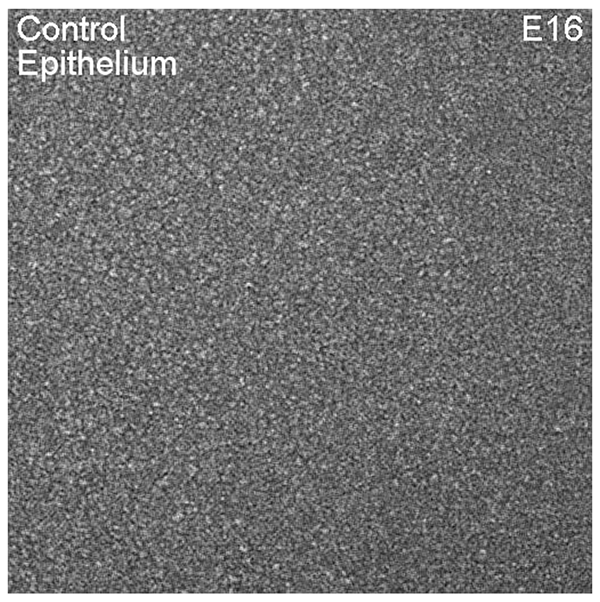


**Supplementary Video 6:** Through-focus SHG collagen dataset of the collagen organization, going from the epithelium layer towards the endothelium layer, in the embryonic corneal stroma at E16.


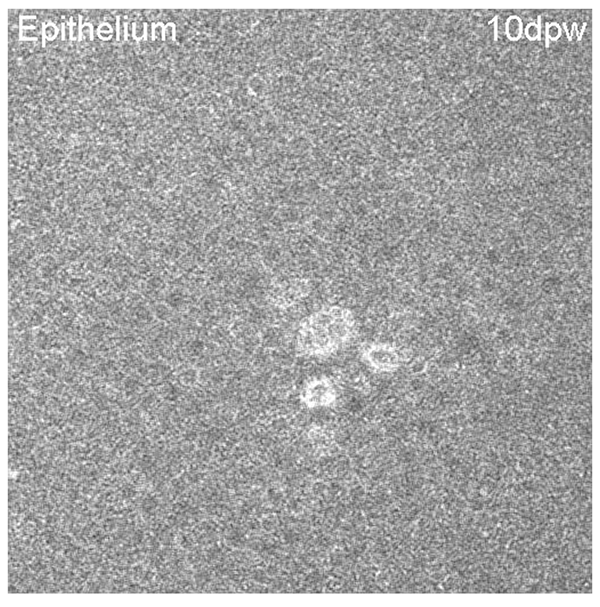


**Supplementary Video 7:** Through-focus SHG collagen dataset of the collagen organization, going from the epithelium layer towards the endothelium layer, at the central wound (C) at 10dpw.


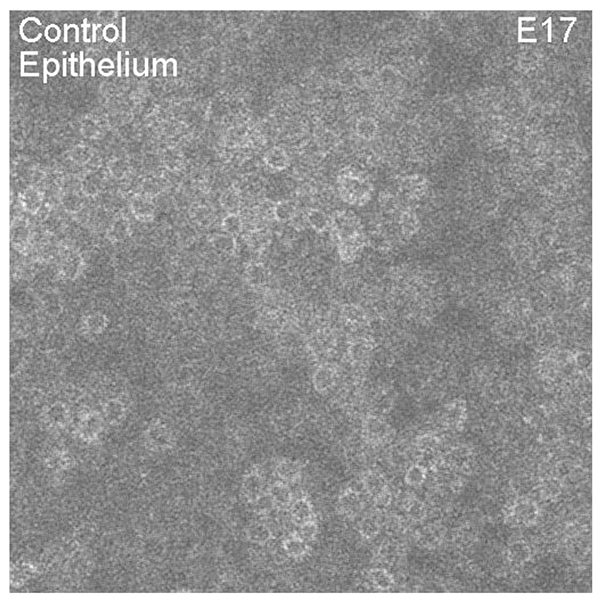


**Supplementary Video 8:** Through-focus SHG collagen dataset of the collagen organization, going from the epithelium layer towards the endothelium layer, in the embryonic corneal stroma at E17.


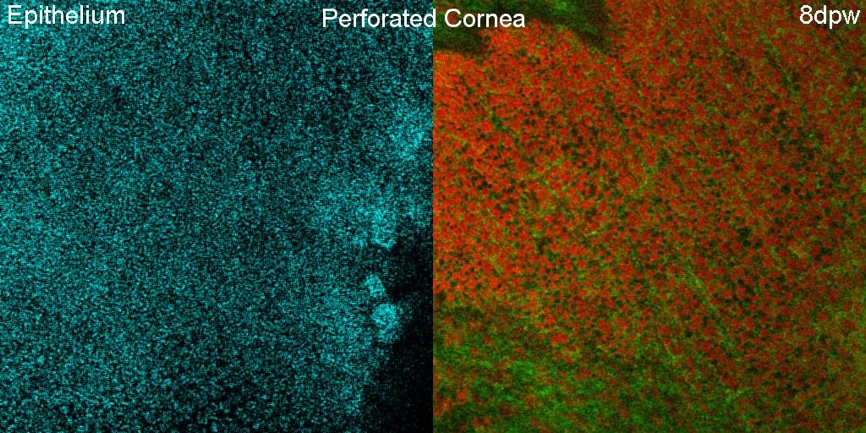


**Supplementary Video 9:** Through-focus SHG collagen dataset of the collagen organization, going from the epithelial layer towards the endothelial layer in the perforated embryonic cornea at 8 dpw (left panel). Through-focus dataset of the nuclei (red) and actin (green) of the same region in the perforated embryonic cornea at 8 dpw.
